# Women’s Word Use in Pregnancy: Associations With Maternal Characteristics, Prenatal Stress, and Neonatal Birth Outcome

**DOI:** 10.3389/fpsyg.2018.01234

**Published:** 2018-07-24

**Authors:** Jessica Schoch-Ruppen, Ulrike Ehlert, Franziska Uggowitzer, Nadine Weymerskirch, Pearl La Marca-Ghaemmaghami

**Affiliations:** ^1^Department of Clinical Psychology and Psychotherapy, University of Zurich, Zurich, Switzerland; ^2^University Research Priority Program – Dynamics of Healthy Aging, University of Zurich, Zurich, Switzerland; ^3^School of Social Work, Institute for Integration and Participation, University of Applied Sciences and Arts Northwestern Switzerland, Olten, Switzerland

**Keywords:** pregnancy, prenatal stress, psychological well-being, birth outcome, word choice, LIWC

## Abstract

**Background:** Experiencing high levels of stress during pregnancy can impair maternal well-being and fetal development. Consequently, unbiased assessment of maternal psychological state is crucial. Self-report measures are vulnerable to social desirability effects. Thus, implicit measures, such as word choice analysis, may offer an alternative.

**Methods:** In this longitudinal online-study, 427 pregnant women described their emotional experiences in writing and additionally responded to self-report questionnaires assessing symptoms of prenatal stress and depression. The written texts were analyzed with a computerized text analysis program. After birth, 253 women provided information on birth outcome.

**Results:** Word use differed significantly depending on maternal socioeconomic (e.g., marital status) and pregnancy-related characteristics (e.g., parity). Prenatal stress and depressive symptoms were associated with more frequent use of negative emotion words and words of anxiety, as well as with less first-person plural, but not singular pronoun use. Negative emotion and cognitive mechanism words predicted birth outcome, while self-report measures did not.

**Conclusion:** In addition to self-report measures, word choice may serve as a useful screening tool for symptoms of depression and stress in pregnant women. The findings on pronoun use may reflect women’s changing experience of self-identity during the transition to motherhood.

## Introduction

Pregnancy and the transition to motherhood is characterized by physiological, psychological, and social changes (Hobbs and Cole, 1976; Yali and Lobel, 1999). The attempt to adapt to these multifaceted changes may result in prenatal stress (La Marca-Ghaemmaghami and Ehlert, 2015). In fact, a majority of women report experiencing psychosocial stress during pregnancy(Woods et al., 2010). Some women may go on to develop negative affect states such as anxiety or depressive symptoms. Prevalence rates for mental health problems during pregnancy vary between studies. Prenatal depressive symptoms are present in approximately 16–37% of all pregnant women and about 5–12% fulfill the criteria for major depression (Lee et al., 2007; Melville et al., 2010; Fall et al., 2013; Lara et al., 2015). Feelings of anxiety seem to occur even more frequently ranging from 14 to 54% (Heron et al., 2004; Lee et al., 2007; Rubertsson et al., 2014). The prevalence rate for anxiety disorders during pregnancy is 13% (Vesga-López et al., 2008). If not adequately treated, prenatal psychological problems may persist throughout pregnancy and into the postpartum period (Grant et al., 2008).

Prenatal stress may not only decrease pregnant women’s well-being, but also can negatively affect fetal development and neonatal birth outcome. For instance, maternal depressive symptoms have been found to be associated with lower neonatal birth weight (Goedhart et al., 2010; Dunkel Schetter and Lobel, 2012) and preterm delivery (i.e., birth before 37 completed week’s gestation) (Grote et al., 2010). Pregnancy anxiety is a specific form of anxiety, which reflects a pregnant woman’s extensive worries about the well-being of her unborn child and fears associated with prenatal medical care, birth, and motherhood. This type of anxiety shows strong links to shorter gestational length (Roesch et al., 2004; Dunkel Schetter and Glynn, 2011) and was found to be a risk factor for preterm birth (Dole et al., 2003; Mancuso et al., 2004; Kramer et al., 2009). However, contradictory findings on the effect of psychological symptoms on birth outcome have been reported as well. For instance, two large population-based studies found no associations between mental health symptoms and neonatal birth outcome (Andersson et al., 2004; Bödecs et al., 2010). Grote et al. (2010) argue that these divergences are likely due to methodological issues such as choice of assessment method or confounding factors. Most studies investigating the consequences of prenatal stress rely on self-report measures by pregnant women as indicators of their mental health status (Lobel, 1994). Yet, self-report questionnaires can elicit socially desirable response behavior, particularly with regard to sensitive topics (King and Bruner, 2000). Consequently, results may be biased with respect to accuracy and completeness of answers (Deshields et al., 1995; Taylor et al., 2006). Thus, self-report measures tend to impair research validity and are often considered a research limitation (Fisher, 1993; Skouteris et al., 2009). Pregnant women oftentimes mention fear of judgment and other negative consequences as barriers to fully disclose their mental health issues to their health care provider (Byatt et al., 2013; Kingston et al., 2015). If pregnant women’s subjective experiences differ substantially from society’s mainly positive view of pregnancy or their own self-image, they might experience feelings of guilt and shame. They could then withhold their problems, and present an image of themselves and of their pregnancy that is more consistent with their own as well as societal expectations (Johnson and Fendrich, 2005). This is in line with findings showing that most psychological difficulties experienced by pregnant women remain unspoken and therefore, untreated (Hatton et al., 2007; Vesga-López et al., 2008; World Health Organization the United Nations Population Fund [WHO], 2008). However, early detection and treatment of mental health problems and stress overexposure are essential to ensure pregnant women’s well-being as well as the healthy development of the child *in utero*.

In order to bypass social desirability or self-presentation biases when assessing sensitive topics, researchers in other fields of psychology and social sciences have turned to implicit measures (Pressman and Cohen, 2007; Hussy et al., 2010; Brönnimann et al., 2013). Particularly, analyzing word use is a promising way to gain insight into psychosocial processes while avoiding response bias in self-reports (Mehl and Pennebaker, 2003; Pennebaker, 2011). The majority of our language is made up of content words such as nouns and verbs which we use to communicate *what* we think (Chung and Pennebaker, 2011). Function words, such as pronouns, form the second group of linguistic characteristics. Even though they only make up about 1% of our natural language, they are closely related to psychosocial processes and describe *how* we think about ourselves and our surroundings (Miller, 1995; Tausczik and Pennebaker, 2010). Accordingly, studies have found individual differences in word use depending on age (Pennebaker and Stone, 2003), gender (Mehl and Pennebaker, 2003), or personality (Hirsh and Peterson, 2009). Moreover, word choice is correlated with an individual’s mental health status (Rude et al., 2004). Studies have consistently confirmed depressive patients’ increased use of first-person singular pronouns reflecting a stronger self-focus compared to non-depressed individuals (Weintraub, 1981; Rude et al., 2004). Depression is further characterized by a more frequent use of negative emotion words (Rude et al., 2004; Ramirez-Esparza et al., 2008). Similarly, the experience of acute stress is reflected in higher use of negative emotion words (Liehr et al., 2004). Unlike the greater self-focus in depression, acute stress results in increased collective orientation, marked by a higher use of first-person *plural* pronouns (Stone and Pennebaker, 2002; Liehr et al., 2004). Apart from reflecting mental health status, word use has also been found to be a predictor for psychotherapy outcome. Lower post-therapy depressive symptoms were related to a decrease in first-person singular pronouns and an increase in positive emotion words during therapy (Pulverman et al., 2015). Studies have provided further evidence for the predictive value of word choice for physical health outcomes. For example, negative emotion words were identified as risk factors for heart disease mortality whilst positive emotion words were found to be protective (Eichstaedt et al., 2015).

Analysis of word use in pregnant women could provide a unique perspective into their emotional experience of pregnancy. Surprisingly, only one study to date has investigated the association between linguistic characteristics and pregnancy in the broader sense: In this retrospective study, parents completed an online questionnaire after the birth of their child and were asked to write about their experience of trying to conceive (Sweeny et al., 2015). The parents’ use of negative emotion words was positively associated with the level of self-reported anxiety during that period. Parents using more first-person plural pronouns reported less anxiety and rumination, while first-person singular pronouns were positively associated with rumination. Moreover, mentioning words targeted at cognitive processes (e.g., *because, think*) were negatively correlated with both anxiety and rumination (Sweeny et al., 2015). Having previously experienced a miscarriage was significantly related to a more frequent use of negative emotions including anxiety and sad words, but in women only (Nelson et al., 2015). Despite these interesting findings, it has to be kept in mind that the study used a retrospective design which could yield memory bias in parents’ recollections (Sweeny et al., 2015). Furthermore, participants described the time during which they were trying to conceive and not their emotional experiences associated with the pregnancy itself. Ensuring mother’s well-being in pregnancy is important for herself and her child. However, both researchers and health care providers face barriers in the assessment of potential psychological problems and stress overload. Therefore, research on linguistic characteristics in pregnancy is required.

### Present Research

The present study was guided by three main research questions. Since word choice has been found to differ between sociodemographic groups or in terms of health conditions (Mehl and Pennebaker, 2003; Lorenz and Meston, 2012), we first investigated associations between pregnant women’s word use and maternal socioeconomic (e.g., educational qualification) and pregnancy-related characteristics (e.g., gestational age). We expected factors such as parity (nulliparous vs. primiparous/multiparous), gestational age, or the experience of complications during pregnancy to influence a woman’s state of mind and therefore, also her choice of words. In self-report studies, nulliparous women, for example, indicated higher fear of childbirth compared to women who already have given birth to a child (Rouhe et al., 2009). Also, pregnant women’s perception of stress seems to decrease with advancing gestation (Glynn et al., 2004). Analyses for this first research question were mainly exploratory in nature since there are hardly any studies on associations between word use and maternal socioeconomic and pregnancy-related characteristics to base our hypotheses on.

Second, this study set out to replicate findings on the relationship between self-reported psychological well-being and word use obtained from other study populations in a sample of pregnant women. We hypothesized prenatal depressive symptoms to be positively associated with the use of first-person singular pronouns and negative emotion words (Rude et al., 2004). We formulated a second analogous hypothesis for prenatal stress, i.e., prenatal stress is related to higher use of first-person singular pronouns and negative emotion words.

Third, we explored the relationship between word use and neonatal birth outcome above and beyond common control variables and self-report measures of the experience of stress and depressive symptoms. Following results from other studies, we expected greater self-focus and negative emotionality – as indicators of stress and depressive symptomatology and therefore, decreased psychological well-being – to be related to poorer birth outcome (i.e., a shorter duration of gestation, and lower neonatal birth weight and size) (Dunkel Schetter and Glynn, 2011; Dunkel Schetter and Lobel, 2012). Deriving from studies on expressive writing, we predicted a positive association between a more frequent use of words that reflect cognitive processing (e.g., *think, know*) on birth outcome (Pennebaker et al., 1997).

## Materials and Methods

### Participants and Procedure

The present data stemmed from a larger online research study on the psychological well-being of pregnant women, which was conducted between April 2012 and October 2013. The study was divided into two parts and addressed pregnant women from German-speaking countries of Central Europe (Switzerland, Germany, Austria, the Principality of Liechtenstein, and Luxembourg). Participants were recruited by placing online advertisement (e.g., in pregnancy forums) as well as displaying flyers in gynecologists’ practices and shops selling baby products. The participants were informed about the study’s objectives and their right to withdraw their participation at any time, without any consequences. The women provided informed consent prior to responding to the online questionnaire. The study was conducted in adherence to the regulations of the Ethics Committee of the Faculty of Arts and Sciences of the University of Zurich. The first online assessment (T1) included information on sociodemographic characteristics (e.g., age, marital status) and pregnancy (e.g., whether the pregnancy was planned or not) as well as several psychometric questionnaires related to the women’s psychological well-being (e.g., the experience of prenatal stress and presence of depressive symptoms). Pregnancy-related variables were assessed by single questions, e.g., “Have you experienced any complications in the present pregnancy?” with two response options (yes/no). Category options for sociodemographic variables in the questionnaire are shown in **Table 1**. At the same time, the women were asked to describe their experiences of pregnancy in writing. Upon agreement, we invited the women to a second online assessment approximately 1 month after the expected delivery date (T2) during which they provided information on birth outcome (e.g., gestational length, neonatal weight, and size at birth). From the original sample of 725 pregnant women, we excluded the texts of 294 women, as they used fewer than 40 words. This minimum of 40 words was required to increase the reliability of the results^1^. Another 4 women were excluded due to multifetal pregnancy which could influence their children’s birth outcome (e.g., with regard to gestational length and birth weight). Even though it would be important to investigate these women’s experiences in pregnancy, the small number of cases did not allow us to test corresponding hypotheses and conduct statistical analyses. This resulted in a sample size of *N* = 427 for T1, of which *n* = 253 women responded to T2 as well.

**Table 1:** Descriptive statistics of sample.

Maternal sociodemographic characteristics [% (*N*)]		Pregnancy-related characteristics [% (*N*)]	
Age in years^a^	31.00 (18–43)	Gestational age at T1 in weeks^a^	23.00 (4–41)
Pre-pregnancy weight in kg^a^	62.00 (41.00 – 124)	Parity status	
Highest educational qualification		Nulliparous	53.2 (227)
University degree	32.1 (137)	Primi- or multiparous	46.8 (200)
University of applied sciences degree	31.6 (135)	Planned Pregnancy	
Secondary school with qualification for university entrance	9.8 (42)	Yes	81.5 (348)
Apprenticeship	17.3 (74)	No	18.5 (79)
Secondary school	8.7 (37)	Former pregnancy loss	
No completed degree	0.5 (2)	Yes	25.5 (109)
Relationship status		No	74.5 (318)
Single	1.4 (6)	Pregnancy complications at T1	6.2 (26)
Stable and not cohabiting	6.1 (26)	Yes	22.0 (94)
Stable and cohabiting	92.5 (395)	No	78.0 (333)
Marital status		Pregnancy complications at T2^b,c^	
Not married	28.1 (120)	Yes	30.7 (131)
Married	67.0 (286)	No	69.3 (296)
Civil union	2.1 (9)	Gestational length in weeks^a,b^	39.43 (1.95)
Separated	0.2 (1)	Birth weight in grams^a,b^	3381.33 (531.75)
Divorced	2.3 (10)	Birth length in cm^a,b^	50.27 (2.81)
Widowed	0.2 (1)		

*N = 427. ^a^Values are presented as median (range). ^b^n = 253. ^c^Pregnancy complications at T2 were retrospectively assessed complications women experienced during pregnancy including those already reported in T1.*

### Measures

#### The Psychological Experience of Stress During Pregnancy

Pregnancy-specific stress was assessed with the German version of the Prenatal Distress Questionnaire (Yali and Lobel, 1999; Pluess et al., 2010). The scale consists of 12 items, which measure the concerns and worries of women regarding pregnancy and birth, e.g., *“I am worried about eating healthy foods and a balanced diet for the infant.”* Items are rated on a five-point Likert scale from 1 (not at all worried) to 5 (extremely worried). A Cronbach’s alpha of 0.808 for the present study indicates a good internal consistency of the measure.

#### Symptoms of Depression

The German version of the Edinburgh Postnatal Depression Scale (EPDS) was used to measure depressive symptoms (Cox et al., 1987; Bergant et al., 1998). This is a well-validated self-report questionnaire, often used to evaluate women’s psychological state during pregnancy and the postpartum period (Nast et al., 2013). The women responded to ten items assessing how they felt in the past week using a scale from 1 (*most of the time*) to 4 (*not at all*), e.g., *“I have felt sad or miserable”*. In this study, the scale’s internal consistency was good (α = 0.851).

#### Word Use

Following recommendations by other authors (Pennebaker, 1989; Horn and Mehl, 2004), participants were presented with the following instructions: *“For this study, could you please take some time (about 5 min) to describe your feelings and thoughts regarding this pregnancy? You do not need to worry about spelling, sentence structure, or grammar.”* The resulting texts were analyzed with the Linguistic Inquiry and Word Count (LIWC; Pennebaker et al., 2007). LIWC is a software that uses a word count approach to analyze a written text. The software has an internal dictionary which consists of more than 4500 words and word stems that are presorted into linguistic and psychological categories (Pennebaker et al., 2007). LIWC calculates the frequency of words from the written sample that fall into a given category by comparing each word against its internal dictionary. We applied the German LIWC dictionary which was shown to have equivalently good psychometric properties compared to the original English version (Wolf et al., 2008). Before data analysis, we corrected the texts for spelling errors. On average, women wrote 91.08 words (*SD* = 55.33). Almost 78% (*SD* = 5.43) of all words were recognized by the LIWC dictionary.

Quantity of word categories were computed as the percentage of words in the specific categories related to the total length of the text. Multiple linguistic categories were used for the current study: first-person singular (e.g., I, me) (*M* = 8.97%, *SD* = 4.64) and first-person plural pronouns (e.g., we, us) (*M* = 1.29%, *SD* = 2.24), positive emotion words (e.g., love, nice) (*M* = 4.84%, *SD* = 2.61), negative emotion words (e.g., hate, hurt) (*M* = 3.25%, *SD* = 2.54) including anxiety (e.g., nervous, tense) (*M* = 1.60%, *SD* = 1.71), and sad words (e.g., cry, grief) (*M* = 0.50%, *SD* = 0.92). Additionally, we were interested in the word category cognitive mechanisms (e.g., think, understand) (*M* = 12.04%, *SD* = 3.97), including words of cause (e.g., because, effect), (*M* = 2.09%, *SD* = 1.78), discrepancy (e.g., should, would) (*M* = 2.05%, *SD* = 1.72), insight (e.g., consider, know) (*M* = 2.75%, *SD* = 2.00), inhibition (e.g., constrain, stop) (*M* = 0.35%, *SD* = 0.73), and certainty (e.g., absolutely, sure, always, never) (*M* = 3.37%, *SD* = 2.18).

#### Birth Outcome Measures

The women responded to questions regarding birth outcome (i.e., gestational length, weight and size of the neonate) at T2 and were subsequently asked about the source of this information. Most women (82.2%, *n* = 208) reported that they had obtained the information from medical records and birth certificates. A further 7.91% (*n* = 20 women) described that they had received the information orally from their obstetrician, pediatrician, and/or midwife, 5.93% (*n* = 15) indicated that they remembered it (e.g., “I know it by heart”), and 3.95% (*n* = 10) did not indicate the source of information. Validation studies show excellent agreement between maternal reports in the months after delivery and medical records regarding gestational age at delivery, neonatal birth weight and size (Troude et al., 2008; Bat-Erdene et al., 2013).

### Statistical Analyses

Statistical analyses were performed using the IBM Statistical Package for the Social Sciences (SPSS Version 22 for Windows). Since several linguistic categories were positively skewed, we applied log transformation using the formula ln (score + 1) (Field, 2013; Schultheiss, 2013). Visual inspections and Kolmogorov–Smirnov tests showed that most variables were still not normally distributed, even though the skewness of the data could be improved. Therefore, we further used non-parametric tests for all analyses, e.g., Spearman correlations (Delucchi and Bostrom, 2004). For education, a dichotomized variable was created with women with a University degree classified as 1 for highest educational qualification. Relationship status was dichotomized by assigning the value 1 for all women in a relationship (cohabiting or not). For marital status, two categories were computed with 1 indicating being married at the moment.

Prior to investigating the research questions, we examined potential differences in sociodemographic, and pregnancy-related variables, as well as psychological measure (i.e., experience of stress and depressive symptoms) between women participating at T1 and those participating at both T1 and T2 using Mann–Whitney *U* tests. Pregnant women who participated at both T1 and T2 were later in gestation (*Mdn = 25.00*) compared to women who did not provide data at T2 (*Mdn* = 21.50; *U* = 17633.50, *p* < 0.001) and experienced less prenatal stress (*Mdn* = 6.00) and depressive symptoms (*Mdn* = 12) compared to those who did not respond to T2 (*Mdn* for prenatal stress = 7.00; *U* = 17326.50, *p* = 0.003; *Mdn* for depressive symptoms = 14.00; *U* = 16583.00, *p* = 0.016).

For the first research question, potential associations between maternal sociodemographic and pregnancy-related characteristics and word choice were explored with Spearman correlations. Group differences in word choice were analyzed with Mann–Whitney *U* tests (i.e., group differences in word choice regarding maternal educational qualification, marital status, (un-)planned pregnancy, (nulli-)parity, prior pregnancy loss, and current pregnancy complications). Effect sizes were calculated according to the formula *r* = z / √N and interpreted as small (*r* = 0.1), medium (*r* = 0.3), or large (*r* = 0.5) (Cohen, 1990; Rosenthal, 1991).

With regard to the second research question, the relationship between word choice and maternal psychological state was investigated with partial Spearman correlations. To determine relevant control variables prior to the main statistical analyses, point-biserial correlations between maternal psychological state (i.e., the experience of stress and depressive symptoms during pregnancy) and the following variables were computed: maternal age, education, marital status, relationship status, parity, unplanned pregnancy, pregnancy complications at T1, and gestational week at T1. For prenatal stress, the following variables were significant and therefore, controlled for: maternal age (*r_s_* = −0.272, *p* < 0.001), education (*r_s_* = −0.105, *p* = 0.035), marital status (*r_s_* = −0.142, *p* = 0.004), relationship status (*r_s_* = −0.137, *p* = 0.006), parity (*r_s_* = 0.147, *p* = 0.003), unplanned pregnancy (*r_s_* = 0.124, *p* = 0.13), and gestational week at T1 (*r_s_* = −0.200, *p* < 0.001). With regard to depressive symptoms, maternal age (*r_s_* = −0.153, *p* = 0.002), marital status (*r_s_* = −0.120, *p* = 0.016), unplanned pregnancy (*r_s_* = 0.229, *p* < 0.001), relationship status (*r_s_* = −0.177, *p* < 0.001), and pregnancy complications at T1 (*r_s_* = 0.203, *p* < 0.001) were significant and added as control variables in the relevant analyses.

For the final research question, we ran block-wise multiple regressions to determine the influence of prenatal stress, depressive symptoms, and word choice on birth outcome. In the first block, we entered control variables from sociodemographic and pregnancy-related domains. Again, control variables were determined beforehand with correlation analyses. In addition to the variables described in the previous paragraph, pregnancy complications at T2, maternal pre-pregnancy weight, the newborn’s gender, and gestational length were tested for their potential influence on birth outcome. For gestational length, only gestational age at T1 was significant and chosen as a control variable. For neonatal birth weight and size, gestational length, maternal pre-pregnancy weight, and newborn’s gender were significantly correlated and entered into the respective models as control variables. The second block was composed of self-reported psychometric measures, namely prenatal stress and depressive symptoms. In a third step, word choice was entered into the analyses. Word categories included first-person singular and plural pronouns, positive emotions, negative emotions (including words of anxiety and sadness), and cognitive mechanisms (including words of certainty, insight, discrepancy, and inhibition). A step-wise selection method was applied to the second and third block so that at each step only the variable with the largest significant contribution was entered into the model (Field, 2013). For the results presented here, non-significant variables were dropped and a final block-wise regression was run in which all significant predictors established in the first regression were entered simultaneously.

Statistical significance was defined as *p* < 0.05, and all analyses were two-tailed.

## Results

### Sample Characteristics

The descriptive statistics of the study sample are presented in **Table 1**.

### Is Word Choice Associated With Maternal Sociodemographic and Pregnancy-Related Characteristics?

Concerning maternal sociodemographic characteristics, Spearman correlations indicated that maternal age was associated with fewer anxiety words (*r_s_* = −0.120, *p* = 0.013). Women who had attained a higher educational qualification (*n* = 137) wrote fewer first-person plural pronouns (*U* = 17447.50, *p* = 0.022, *r* = 0.11), but more words of inhibition (*U* = 17831.50, *p* = 0.026, *r* = 0.11) in their texts than women with a lower educational qualification (*n* = 290). Pregnant women who were not in a stable relationship (*n* = 6) used less anxiety words compared to women with a partner (*U* = 656.50, *p* = 0.040, *r* = 0.10; *n* = 421). Married women (*n* = 286) compared to non-married women (*n* = 141) used more words indicating certainty (*U* = 17579.50, *p* = 0.031, *r* = 0.10).

With regard to pregnancy-related characteristics, gestational age was negatively correlated with the use of first-person singular pronouns (*r_s_* = −0.123, *p* = 0.011) and words of insight (*r*_s_ = −0.119, *p* = 0.014). Then, the frequency of negative emotion and sad words was significantly higher in nulliparous women (*n* = 227) compared to women who had given birth before (*U* = 20014.50, *p* = 0.035, *r* = 0.10 and *U* = 19942.50, *p* = 0.008, *r* = 0.13; *n* = 200). Women used significantly fewer words of certainty, if the pregnancy was unplanned (*U* = 11502.50, *p* = 0.023, *r* = 0.11; planned pregnancy: *n* = 348; unplanned pregnancy: *n* = 79). The use of anxiety words was elevated in women who had experienced at least one pregnancy loss in the past (*U* = 15151.50, *p* = 0.047, *r* = 0.10; *n* = 109), while their use of first-person plural pronouns was decreased compared to women who had never had a pregnancy loss before (*U* = 14793.50, *p* = 0.010, *r* = 0.13; *n* = 318). Women experiencing complications in the present pregnancy (*n* = 94) used significantly less first-person singular pronouns (*U* = 13092.00, *p* = 0.015, *r* = 0.12), more negative emotion words (*U* = 13539.00, *p* = 0.045, *r* = 0.21), and fewer words of certainty (*U* = 12886.50, *p* = 0.009, *r* = 0.13) compared to women who did not experiency any pregnancy complications (*n* = 333).

### Does Word Use Reflect Pregnant Women’s Self-Reported Psychological State?

Findings on the analyses of the relationship between maternal psychological symptoms as measured by self-report questionnaires and word choice are reported in **Table 2**.

**Table 2:** Partial Spearman correlation coefficients for the associations between maternal psychological state and word choice.

	1st person singular pronouns	1st person plural pronouns	Positive emotions	Negative emotions	Anxiety	Sadness	Cognitive mechanisms	Certainty	Insight	Discrepancy	Inhibition
Prenatal stress	−0.024	−0.131^∗∗^	−0.085†	0.234^∗∗∗^	0.187^∗∗∗^	0.068	−0.096†	−0.054	−0.059	−0.036	0.075
Depressive symptoms	−0.064	−0.125^∗^	−0.124^∗^	0.280^∗∗∗^	0.160^∗∗^	0.225^∗∗∗^	−0.034	−0.059	0.060	−0.073	0.037

*n = 403 for prenatal stress. Partial Spearman correlations controlled for maternal age, education, marital status, relationship status, parity, unplanned pregnancy, and gestational age at T1. n = 399 for depressive symptoms. Partial Spearman correlations controlled for maternal age, marital status, relationship status, unplanned pregnancy, and pregnancy complications at T1. Significance levels (two-tailed): ^†^p < 0.10, ^∗^p < 0.05, ^∗∗^p < 0.01, ^∗∗∗^ p < 0.001.*

### Is Maternal Word Use During Pregnancy Associated With Neonatal Birth Outcome?

The association between maternal psychological state, word choice and birth outcome was analyzed separately for gestational length, neonatal birth weight, and birth size. Prenatal stress and depressive symptoms assessed using self-report questionnaires did not show any relationship with pregnancy duration and were therefore dropped from the final analysis. For gestational length, including word choice resulted in a significant increase in Δ*R^2^* = 0.04, *F*(3,249) = 6.66, *p* < 0.001. First-person plural pronoun use (*β* = −0.18, *p* = 0.005) as well as negative emotion words (*β* = −0.14, *p* = 0.024) predicted gestational length (see **Table 3**). Hence, women using more first-person plural pronouns (e.g., we) and negative emotion words (e.g., hurt) delivered their babies earlier. In total, the predictors could explain 6% of variance in gestational length.

**Table 3:** Block-wise regression analysis for gestational length.

	Gestational length					
Predictors	*β*	*t*	*p*	*R^2^*	Δ*R^2^*	*F*
*Block 1*				0.03	0.03	8.56^∗∗^
Gestational age T1	0.18	2.93	0.004			
*Block 2*				0.06	0.04	6.66^∗∗∗^
1st ps. plural pronouns	−0.18	−2.83	0.005			
Negative emotion	−0.14	−2.27	0.024			

*n = 253. Significance levels (two-tailed): ^∗∗^p < 0.01, ^∗∗∗^p < 0.001.*

Results of the regression analysis for birth weight are presented in **Table 4**. Self-reported measures of maternal psychological state did not predict birth weight. Entering word choice in the second block led to a significant increase in Δ*R^2^* = 0.01, *F*(5,245) = 40.88, *p* < 0.001. Using more first-person plural pronouns predicted lower birth weight (*β* = −0.11, *p* = 0.035). The final model was able to explain 39% of variance in birth weight.

**Table 4:** Block-wise regression analysis for neonatal birth weight.

	Birth weight					
Predictors	*β*	*t*	*p*	*R^2^*	Δ*R^2^*	*F*
*Block 1*				0.38	0.39	52.27^∗∗∗^
Gestational length	0.60	11.72	0.000			
Pre-pregnancy weight	0.10	2.01	0.046			
Newborn’s gender	0.11	2.22	0.028			
*Block 2*				0.39	0.01	40.88^∗∗∗^
1st ps. plural pronouns	−0.11	−2.12	0.035			

*n = 252. Significance levels (two-tailed): ^∗∗∗^p < 0.001.*

Neonatal size at birth was not predicted by depressive symptoms and prenatal stress, as assessed by self-report. Adding word choice explained significantly more variance, Δ*R^2^* = 0.01, *F*(4,243) = 36.04, *p* < 0.001. Cognitive mechanisms predicted larger birth size (*β* = 0.12, *p* = 0.024). Overall, the predictors explained 36% of variance in birth size. Results are shown in **Table 5**.

**Table 5:** Block-wise regression analysis for neonatal birth size.

	Birth size					
Predictors	*β*	*t*	*p*	*R^2^*	Δ*R^2^*	*F*
*Block 1*				0.35	0.36	45.55^∗∗∗^
Gestational length	0.53	10.20	0.000			
Pre-pregnancy weight	0.17	3.27	0.001			
Newborn’s gender	0.16	3.07	0.002			
*Block 2*				0.36	0.01	36.04^∗∗∗^
Cognitive mechanisms	0.12	2.28	0.024			

*n = 249. Significance levels (two-tailed): ^∗∗∗^p < 0.001.*

## Discussion

### Summary and Discussion of Results

The goal of this study was to explore pregnant women’s word choice, its association with maternal socioeconomic and pregnancy-related characteristics, self-report measures of prenatal stress and depressive symptoms, and its predictive value for pregnancy duration and neonatal birth outcome. Analyses for the first research question yielded that married women used words of certainty more frequently than unmarried women, while single women wrote fewer anxiety words compared to women in a stable relationship. Higher maternal age was associated with fewer anxiety words as well. Word choice also differed depending on pregnancy-related factors, such as nulliparity, former pregnancy loss, or the experience of pregnancy complications. In these cases, women’s texts contained more negative emotion or anxiety words, respectively. Frequency of certainty words was decreased in women with an unplanned pregnancy and in those experiencing pregnancy complications. In the latter, the use of first-person singular pronouns was reduced as well, while women who had experienced a former pregnancy loss used less first-person plural pronouns. If pregnancy was more advanced, pregnant women mentioned fewer first person singular pronouns and words of insight.

For the second research question, we analyzed the relationship between maternal psychological state and word choice. Women reporting increased prenatal stress used more negative emotion words and anxiety words. Similarly, women reporting higher depressive symptoms used fewer positive emotion words, more negative emotion words, including words of anxiety and sadness. In line with the literature, we had expected depressive symptoms in pregnant women to be associated with increased use of first-person singular pronouns. This, however, was not the case. Instead, higher depressive symptomatology and likewise increased prenatal stress were associated with fewer first-person plural pronouns.

Finally, when investigating the association between word use and birth outcome, we found women’s more frequent use of negative emotion words, but surprisingly also more frequent use of first-person plural pronouns to predict shorter gestational length. Likewise, lower neonatal birth weight was predicted by writing more words such as *we* or *our*. Increased birth size, though, was associated with pregnant women’s more frequent use of words relating to cognitive mechanisms.

To date, hardly any study has investigated word use in pregnant women. Thus, the present findings contribute to our advancement of understanding maternal psychological processes during pregnancy and their impact on fetal development. In the following, we will discuss the results based on the literature on lexical choice in other non-pregnant study samples.

The first research question addressed associations between linguistic and maternal sociodemographic as well as pregnancy-related characteristics. Our finding of a higher usage of certainty words in married compared to unmarried women corresponds to results indicating higher pregnancy wantedness in married compared to cohabitating and single women (Santelli et al., 2003; Maxson and Miranda, 2011) and a higher prevalence of a planned pregnancy in married compared to non-cohabitating pregnant women (Wellings et al., 2013). Then, we found higher maternal age to be associated with fewer anxiety words. This is in line with a study by McMahon et al. (2011), in which older pregnant women (>37 years) reported lower symptoms of anxiety and depression and seemed to be more resilient than their younger counterparts. However, this advantage of advanced maternal age may be annuled in older pregnant women who conceive via assisted reproductive technology (McMahon et al., 2011). Surprisingly, in our sample, single pregnant women used fewer anxiety words than the women in a stable relationship. This result should, however, be considered with caution due to the small sample size (*n* = 6) in the group of single pregnant women. Few studies have systematically compared the effect of singlehood, cohabitation, and marriage on maternal psychological wellbeing in pregnancy – even less so with regard to word use. Maternal singlehood in pregnancy has been linked with an increased risk for the experience of postpartum depressive symptoms (Lara et al., 2016). Moreover, the prevalence of mental health problems seems to be increased in single mothers compared to mothers who have a partner (Crosier et al., 2007). This difference was explained by higher financial problems and decreased social support in single mothers.

With regard to pregnancy-related characteristics, the positive associations between negative emotion words and negative experiences such as pregnancy complications were to be expected, since negative experiences or states (e.g., psychiatric disorders) tend to be reflected in a more negative word use in non-pregnant participants (Rude et al., 2004; Orsillo et al., 2004; Minor et al., 2015). Furthermore, similar to marital status, women facing greater insecurity in pregnancy, such as when they had not planned their current pregnancy, or when they were confronted with obstetrical risks, expressed these concerns in their written texts by using less words indicating certainty such as *absolutely, sure*, *always*, or *never*. Similarly, in a study with breast-cancer patients, higher use of linguistic uncertainty was related to lower well-being and greater depression (Cordova et al., 2001).

The results for first-person singular and plural pronouns are somewhat contradictory to the literature. A more frequent use of first-person singular pronouns (e.g., I, me) has been associated with impaired mental health, such as increased depressive symptoms (Stirman and Pennebaker, 2001; Rude et al., 2004). Accordingly, one would assume that the experience of obstetric complications, which are known to increase the risk of depressive symptoms (Brandon et al., 2008; Byatt et al., 2013), is linked with a more frequent use of first-person singular pronouns. However, in our study, women with pregnancy complications wrote less first-person singular pronouns compared to women without obstetric complications. This might possibly indicate that when women worried about the health of their unborn child, their focus shifted away from themselves. A lower usage of first-person singular pronouns, however, was associated with a more advanced pregnancy, indicating a decrease in self-focus with impending birth.

Plural pronouns, under certain circumstances, appear to be a marker of shared identity, closeness, and affiliative motivation (Slatcher, 2009; Chung and Pennebaker, 2011). After the terror attack on the World Trade Center Towers on September 11th 2001, researchers noted increases in plural pronoun use suggesting increased collective orientation in this time of stress (Mehl and Pennebaker, 2003; Liehr et al., 2004). Some researchers suggest that referring to ourselves as a dyad or group has adaptive psychological consequences (Kelley and Thibaut, 1978; Zimmermann et al., 2013). Therefore, acute stress seems to increase social connectedness and the use of plural pronouns. In the long term, this collective orientation may improve mental health and well-being. Accordingly, Frost (2013) found participants using more first-person plural pronouns to report fewer depressive symptoms and higher psychological well-being. Similarly, in the texts of parents who retrospectively described their thoughts and feelings about the time when they were trying to conceive, more first-person plural pronouns were associated with less anxiety and rumination (Sweeny et al., 2015). In the present study though, women who had experienced a former pregnancy loss, wrote fewer first-person plural pronouns. This could indicate a lack of social connectedness. Several qualitative interview studies show that women experiencing a miscarriage tend to suffer in silence and feel disconnected (Adolfsson et al., 2004; MacWilliams et al., 2016). Furthermore, they may regard their pregnancy loss as a personal failure accompanied by feelings of their own guilt (Wong et al., 2003; Adolfsson et al., 2004). It could also be argued that women who have had to deal with a pregnancy loss in their past are emotionally distancing themselves from their unborn child due to fears of another pregnancy loss (Adolfsson et al., 2012). However, studies on the impairment of prenatal attachment in subsequent pregnancies after a pregnancy loss provided mixed results (Tsartsara and Johnson, 2006; Alhusen, 2008). In sum, pregnant women differ from other samples in regard to their use of singular and plural pronouns. Future research is needed to understand the mechanisms of these associations more clearly.

For the second research question on word use and maternal psychological state, we expected to replicate consistent findings from previous research in our sample of pregnant women. However, our hypotheses were only partially supported. We found worse self-reported maternal psychological state, that is higher prenatal stress and depressive symptoms, to be reflected in a higher frequency of negative emotion words including words of anxiety and sadness. This is similar to other studies, in which negative emotion words were positively related to depression or the experience of sexual abuse (Ramirez-Esparza et al., 2008; Lorenz and Meston, 2012). Yet unexpectedly, we did not find the negative relationship between maternal psychological state and first-person singular pronouns (Stirman and Pennebaker, 2001; Rude et al., 2004). In the present study, instead, women experiencing increased prenatal stress and depressive symptoms used first-person *plural* pronouns *less* frequently. Comparable results have been obtained in a study by Frost (2013) in which lower psychological well-being was associated with lower use of first-person plural pronouns. Even though acute stress is supposed to increase collective orientation and have benefits for well-being (Mehl and Pennebaker, 2003; Zimmermann et al., 2013), the experience of prolonged stress and depressive symptoms seems to have the opposite effect by narrowing our focus on the self. However, instead of an increase in single pronouns as in other studies (Rude et al., 2004), we observed a decrease in plural pronouns in pregnant women with lower psychological well-being. Possibly, and under certain circumstance, decreased psychological well-being in pregnant women is not reflected by a stronger focus on the single self (*I*, *mine*), but by decreased attention to a shared self (*us*, *our*). Pregnancy can evoke changes in women’s identity and the transition to motherhood expands women’s self (Laney et al., 2014, 2015). Therefore, women referring to themselves as a family instead of an individual could be interpreted as a sign of collective orientation, which may have beneficial, adaptive psychological consequences. Hence, women’s increased or decreased use of first-person *plural* pronouns might be more indicative of pregnant women’s psychological state than their choice of *singular* pronouns.

Our results suggest that word use is indeed associated with self-reported psychological symptoms in pregnancy. Nevertheless, we could also show that findings from previous studies cannot easily be generalized to pregnant women. They might differ in word choice from other participants because they experience changes in the representation of their self during pregnancy, which is reflected in changes in their word use. Therefore, future studies should investigate pregnant women’s word use and its associations with their psychological state in more depth, particularly with regard to the use of first-person singular and plural pronouns.

For the final research question, we explored the relationship between word use and birth outcome variables above and beyond that of self-reported prenatal stress and depressive symptoms and common control variables. Interestingly in this study, maternal self-reported psychological state was not related to any birth outcome measure in the first place. Therefore, this hypothesis could not be tested as originally planned. The lacking association, however, corresponds to studies reporting no significant associations between women’s psychological symptoms or psychiatric disorders and children’s birth outcome (Andersson et al., 2004; Bödecs et al., 2010). Still, several studies found evidence for the influence of self-reported depressive symptoms or prenatal anxiety on adverse birth outcome (Grote et al., 2010; Dunkel Schetter and Lobel, 2012). This divergence may be explained by socially desirable response behavior that biased the self-reported measures of women’s mental health. Alternatively, pregnant women might want to see themselves in a different way compared to their true feelings. Therefore, in self-reports, they might present an image of themselves that is consistent with their own, more positive self-image in order to protect their self. Relying on self-report measures bears the risk of under-identification and under-treatment of psychological problems in pregnancy due to feelings of guilt or shame pregnant women might experience (World Health Organization the United Nations Population Fund [WHO], 2008). Implicit measures allow for an additional, less biased perspective and insight into women’s psychological state during pregnancy. In the present study, this assumption is supported by several linguistic categories that were indeed found to be significant predictors of birth outcome. Negative emotion words predicted shorter gestational length, suggesting that a negative emotional state could have a negative influence on the course of pregnancy and on fetal development. Since this relationship could not be found for women’s self-reported psychological state, the analysis of word choice such as higher use of negative emotion words could be an effective screening tool for lower well-being in pregnancy. Experiencing frequent negative emotional states and excessive stress during pregnancy has been associated with a dysregulated maternal hypothalamic-pituitary-adrenal (HPA) axis and increased circulating cortisol levels in the maternal blood (La Marca-Ghaemmaghami and Ehlert, 2015). Cortisol can pass the placental barrier more easily under maternal chronic stress conditions and exert adverse effects on the development of the fetus (Ghaemmaghami et al., 2014; La Marca-Ghaemmaghami et al., 2015, 2017).

The findings with regard to the use of first-person plural pronouns and birth outcome are again somewhat contradictive. Our second research question revealed associations between lower psychological well-being and less use of first-person plural pronouns. Based on these results, we would have expected a positive influence of first-person plural pronouns (e.g., *we*) on birth outcome, as it seemed to reflect higher maternal well-being. However, more frequent use of first-person plural pronouns negatively predicted gestational length and neonatal birth weight. Since studies found an increase in plural pronouns after a stressful event, we could interpret pregnant women’s increased use of first-person plural pronouns to be an indicator of experienced stress in pregnancy that could not be detected with self-report measures. Possibly, higher usage of we-pronouns may reflect an increased experience of social support in response to an increased experience of prenatal stress. From this point of view, the negative association between plural pronouns and birth outcome is more coherent. It is noteworthy, though, that studies have shown first-person plural pronouns to have other meanings besides a shared sense of identity (Tausczik and Pennebaker, 2010). Further research is needed to clarify the mechanisms behind the use of plural pronouns during pregnancy and in general, particularly how they are affected by different forms of stress or the experience of social support and their relevance for well-being and health.

The amount of cognitive mechanism words was a predictor of higher birth size. This linguistic category is a marker of cognitive activity and the active construction of meaning (Graham et al., 2009; Pennebaker et al., 2015). It has been associated with better physical health (Pennebaker et al., 1997; Low et al., 2006). For example, caregivers who wrote about their partner’s suffering showed higher heart rate reactivity when their texts contained fewer words of cognitive processing (Monin et al., 2012). Studies on writing as a therapeutic technique showed that improvement in therapy was associated with patients using more cognitive words over the course of time (Pennebaker et al., 1997; Campbell and Pennebaker, 2003). In conclusion, thinking and processing the manifold events in pregnancy could be a form of emotion regulation and could predict healthier birth outcome through a stress-protective biological pathway. More frequent use of cognitive mechanism words has been associated with decreased anxiety and rumination in couples who described their experience of trying to conceive (Sweeny et al., 2015).

In order to ensure pregnant women’s well-being and a healthy development of their child, prenatal mental health care needs to be incorporated into traditional health care services during pregnancy (World Health Organization the United Nations Population Fund [WHO], 2008). However, psychological symptoms in pregnancy often remain unspoken due to feelings of guilt and shame resulting in biased information. Our results suggest that analyzing word choice, particularly regarding the use of sad words and words of certainty might serve as a helpful tool in screening for psychological symptoms in pregnancy and reduce the problems associated with social desirability and positive self-representation.

### Limitations, Strengths, and Future Research

Several limitations of this study need to be discussed. Participants were asked to spend about 5 min writing their response to the open-ended question. However, text samples were provided online without controlling for whether the women stuck to this time frame. Consequently, most participants provided relatively short text samples. Many studies using the word count approach had participants write for a certain amount of time or space, such as writing for 20 min on an A4-paper (Lorenz and Meston, 2012; Kim et al., 2015). However, there have been studies using shorter text samples, which yielded reliable results (Holtgraves, 2011; Tumasjan et al., 2011). For the current study, reliability of analysis was enhanced by only including texts with a minimum of 40 words. Nevertheless, future studies might consider setting requirements such as a minimum amount of writing time or space. Furthermore, by assessing several text samples throughout pregnancy, researchers would be able to investigate changes in word choice during this emotional period in a woman’s life. Similarly, maternal psychological well-being was measured only once during pregnancy. In the present study, depressive symptoms and prenatal stress had no impact on birth outcome. However, research has shown variability in these measures depending on prenatal stage (Teixeira et al., 2009). Hence, several measurement time points during pregnancy could provide a more detailed picture of women’s mental well-being and its relation to linguistic variables and birth outcome.

Participating women were of different gestational ages at the first measurement time point, resulting in a large variability. However, this circumstance was purposely taken into account in order to explore whether maternal well-being and word use might differ depending on the time point of pregnancy. Indeed, maternal prenatal stress at T1 was negatively associated with gestational age, suggestive of decreased stress with advancing pregnancy – a finding which is in line with results from previous studies (e.g., Glynn et al., 2004; Lübke et al., 2017). And, more frequent use of first-person singular pronouns was associated with a less advanced pregnancy which may indicate changes in women’s representation of self with advancing pregnancy in such a way that their focus on themselves decreases with approaching birth.

Another limitation concerns the high drop-off rate observed at T2. Women dropping out of the study after T1 were earlier in their pregnancy at T1 and seemed to be experiencing more stress and depressive symptoms. These women may have been less motivated to respond to our questions at T2 and might have decided to discontinue their participation.

Also, we did obtain a relatively healthy birth sample. Most pregnancies were of average gestational length and most newborns were of normal birth weight and size. Only 7.9% (20) of the infants were born preterm and only 4.8% (12) had low birth weight below 2500 g. Therefore, there might not have been enough variability in the outcome variables for our analyses. However, even in the normal range, lower birth weight is a risk factor for the development of diseases in adulthood (Harris and Seckl, 2011). Also, the prevalence rates of preterm birth in European countries range between 5.3 and 11.4 per 100 life births (Lisonkova et al., 2012), and according to estimations by the United Nations Children’s Fund and World Health Organization (2004) 6.4% of all infants are affected by low birth weight (<2500 g).

The effect sizes in the present study were small and may limit the clinical importance of the results, such as the predictive value of word choice for birth outcome. However, the present effect sizes are comparable to other studies on word choice (Pennebaker and King, 1999; Newman et al., 2003, 2008; Yarkoni, 2010). Nevertheless, more research on this topic is needed to further confirm our results.

Despite these shortcomings, the present study is strengthened by its study design. A major advantage is the application of a multi-method approach to answer our research questions. By combining a cross-sectional and longitudinal study design, we were able to analyze relationships between word choice and variables in pregnancy as well as to determine the predictive value of word choice for birth outcome. Moreover, we combined different assessment methods including self-reported and objective data as well as implicit measures. This offered a unique perspective on women’s psychological state of mind. Our approach allowed us to investigate the independent contribution of word choice to birth outcome while controlling for self-reported maternal psychological state. The study is further strengthened by the inclusion of several control variables such as pregnancy complications.

## Conclusion

It is essential for both research and clinical practice to be able to accurately assess pregnant women’s psychological well-being in order to identify those women at risk for prenatal stress overexposure. Explicit assessment methods are often limited by social desirability or self-presentation bias. The additional use of implicit measures such as word choice may increase measurement accuracy and ensure with greater reliability that pregnant women in distress receive psychological care rapidly. Research efforts have to be intensified in order to gain a better understanding of the psychological dimensions of women’s word use during pregnancy and assess the advantages of word choice analyses as an additional screening tool for impaired psychological well-being.

## Availability of Data and Material

The datasets analyzed during the current study are not publicly available due to terms of consent to which the participants agreed, but are available from the corresponding author upon reasonable request.

## Ethics Statement

The study was carried out in accordance with the regulations of the Ethics Committee of the Faculty of Arts and Sciences of the University of Zurich. All participants provided informed consent.

## Author Contributions

UE and PLM-G designed the study and were involved in the interpretation of the data. PLM-G oversaw the recruitment process and the statistical analyses. FU and NW helped in the recruitment of the participants. JS-R prepared the LIWC text samples, was responsible for data analysis, and drafted the manuscript. All authors reviewed, revised, and provided approval of the final version of the manuscript.

## Conflict of Interest Statement

The authors declare that the research was conducted in the absence of any commercial or financial relationships that could be construed as a potential conflict of interest.

## Footnotes

1 In order to determine the best threshold for the present linguistic analyses, we contacted Professor James Pennebaker, co-developer of the LIWC analysis program which was applied in this study. According to Professor Pennebaker (personal communication, May 28, 2013), the threshold depends on the number of texts available. He suggested a 40-word threshold for a total number of 100 texts, a larger threshold if fewer texts had been available.

## Abbreviations

APA: American Psychological Association
EPDS: Edinburgh Postnatal Depression Sale
LIWC: Linguistic Inquiry and Word Count
SPSS: Statistical Package for the Social Sciences
